# Precision Medicine Assessment of the Radiographic Defect Angle of the Intrabony Defect in Periodontal Lesions by Deep Learning of Bitewing Radiographs

**DOI:** 10.3390/bioengineering12010043

**Published:** 2025-01-08

**Authors:** Patricia Angela R. Abu, Yi-Cheng Mao, Yuan-Jin Lin, Chien-Kai Chao, Yi-He Lin, Bo-Siang Wang, Chiung-An Chen, Shih-Lun Chen, Tsung-Yi Chen, Kuo-Chen Li

**Affiliations:** 1 Ateneo Laboratory for Intelligent Visual Environments, Department of Information Systems and Computer Science, Ateneo de Manila University, Quezon City 1108, Philippines; pabu@ateneo.edu; 2 Department of Operative Dentistry, Taoyuan Chang Gung Memorial Hospital, Taoyuan City 33305, Taiwan; louiszzzzz@cgmh.org.tw; 3 Department of Program on Semiconductor Manufacturing Technology, Academy of Innovative Semiconductor and Sustainable Manufacturing, National Cheng Kung University, Tainan City 701401, Taiwan; m28121562@gs.ncku.edu.tw; 4 Department of Electronic Engineering, Chung Yuan Christian University, Taoyuan City 32023, Taiwan; s11026208@cycu.edu.tw (C.-K.C.); s11026241@cycu.edu.tw (Y.-H.L.); s11026215@cycu.edu.tw (B.-S.W.); chrischen@cycu.edu.tw (S.-L.C.); 5 Department of Electrical Engineering, Ming Chi University of Technology, New Taipei City 243303, Taiwan; 6 Department of Electronic Engineering, Feng Chia University, Taichung City 40724, Taiwan; tsungychen@fcu.edu.tw; 7 Department of Information Management, Chung Yuan Christian University, Taoyuan City 320317, Taiwan

**Keywords:** convolutional neural network, image detection, image enhancement, machine learning, radiographic defect angle, intrabony defect

## Abstract

In dental diagnosis, evaluating the severity of periodontal disease by analyzing the radiographic defect angle of the intrabony defect is essential for effective treatment planning. However, dentists often rely on clinical examinations and manual analysis, which can be time-consuming and labor-intensive. Due to the high recurrence rate of periodontal disease after treatment, accurately evaluating the radiographic defect angle of the intrabony defect is vital for implementing targeted interventions, which can improve treatment outcomes and reduce recurrence. This study aims to streamline clinical practices and enhance patient care in managing periodontal disease by determining its severity based on the analysis of the radiographic defect angle of the intrabony defect. In this approach, radiographic defect angles of the intrabony defect greater than 37 degrees are classified as severe, while those less than 37 degrees are considered mild. This study employed a series of novel image enhancement techniques to significantly improve diagnostic accuracy. Before enhancement, the maximum accuracy was 78.85%, which increased to 95.12% following enhancement. YOLOv8 detects the affected tooth, and its mAP can reach 95.5%, with a precision reach of 94.32%. This approach assists dentists in swiftly assessing the extent of periodontal erosion, enabling timely and appropriate treatment. These techniques reduce diagnostic time and improve healthcare quality.

## 1. Introduction

Periodontal disease is a common oral health issue caused by dental plaque, leading to chronic inflammation and potential tooth loss if untreated [1,2]. The disease is driven by pathogenic microorganisms like Porphyromonas gingivalis and Fusobacterium nucleatum, which trigger immune responses, causing tissue damage, and are linked to systemic diseases such as cardiovascular disease and diabetes [3,4]. Diagnosis typically involves clinical examination and X-rays to assess periodontal pockets and alveolar bone resorption, guiding appropriate treatment, though recurrence is possible [5]. In evaluating periodontal disease conditions, the radiographic defect angle of the intrabony defect (intrabony defect) refers to the angle formed by bone loss as observed in radiographic images. It is commonly used in the diagnosis and assessment of periodontal disease. This angle evaluates the severity and morphology of intrabony defects, which are vertical bone resorptions associated with periodontal disease. These defects occur within the bone structure surrounding the teeth and typically extend apically from the alveolar crest toward the tooth root apex.

In view of the above issues, the objective of this study is to develop an image detection system. This system utilizes deep learning and image processing technology to assist doctors in assessing the severity of intrabony defects. P. Cortellini et al. [6] studied intrabony defects of varying angles in traditional medical diagnosis. They evaluated the improved clinical attachment level (CAL) after treatment with bone graft material and guided tissue regeneration (GTR). The study found that narrow-angle defects (<25 degrees) exhibited significantly more significant CAL gains following GTR treatment compared to wide-angle defects (>37 degrees), indicating a better prognosis for narrow-angle defects in regenerative procedures. In contrast, wide-angle defects showed limited CAL improvement, suggesting lower regenerative potential. Therefore, clinicians should tailor their treatment plans based on defect angles and consider additional techniques when treating wide-angle defects to optimize outcomes. Additionally, research by P. Eickholz et al. [7] demonstrated a clear association between periodontal disease and intrabony defects, highlighting the clinical significance of the 37-degree angle. This angle is an essential parameter for assessing the morphology of intrabony defects and predicting treatment outcomes. Similarly, E. Tsitoura et al. [8] found that defects with angles less than 37 degrees respond more favorably to treatment, while those with angles greater than 37 degrees indicate poorer outcomes.

Recent studies have demonstrated the effectiveness of machine learning in dental imaging, including using convolutional neural network (CNN) for classifying molar positions [9], assisting tartar diagnosis with deep learning [10], improving diagnostic accuracy in detecting lesions with fast R-CNN [11], and measuring periodontal injury [12]. CNNs have also been applied for detecting dental roots, endodontically treated teeth, and implants [13], identifying apical lesions [14]. Vertical bitewing radiography (BW image) is crucial for diagnosing periodontal disease, offering more precise imaging. H. Jayasinghe et al. [15] used Mask R-CNN technology, which significantly enhances diagnostic accuracy in detecting dental lesions, along with a periodontal disease prediction model that achieved 75% accuracy. L. M. Leo et al. [16] demonstrated that CNN technology can effectively identify periodontal disease. By applying median filtering, the accuracy was significantly improved to 96.15%, compared to 85.07% without filtering. This highlights the importance of preprocessing techniques in enhancing model performance for medical imaging tasks. Among the you only look once (YOLO) models, A. Aboah et al. [17] showed that YOLOv8 has a better mean average precision(mAP) performance than YOLOv5 and YOLOv7. K.-C. Li et al. [18] showed how pre-trained CNN architectures, especially for tooth position recognition and periodontal disease detection, reached 91.13% accuracy. Bilateral filtering and other image enhancement methods improve image quality and recognition rates [19,20,21,22,23]. P. Hoss et al. [24] employed five different CNN models for cross-validation, achieving an accuracy of up to 84.8%. Using the VGG model, V. Majanga et al. [25] demonstrated how a maximum accuracy of 83% was achieved in identifying four types of alveolar bone loss, with augmentation enhancing accuracy.

Therefore, this study focuses specifically on assisting dentists in determining whether an intrabony defect is greater or less than 37 degrees using image processing techniques combined with deep learning. In terms of research, this study refers to the research by [18] to enhance the final accuracy of the model, which emphasizes that adaptive bilateral filtering can adjust to the range of offsets and widths, smooth noise, and enhance edges and textures in images. Additionally, this study uses morphological operations such as opening and closing, which involve dilation and erosion to remove noise or fill in continuous areas in the image. Given the need for continuous line adjustments in image processing, closing operations were adopted. The proposed system aims to provide a targeted auxiliary tool for the assessment of intrabony defects, assisting dentists in diagnosis and treatment planning while reducing their workload.

## 2. Materials and Methods

**Figure 1:**
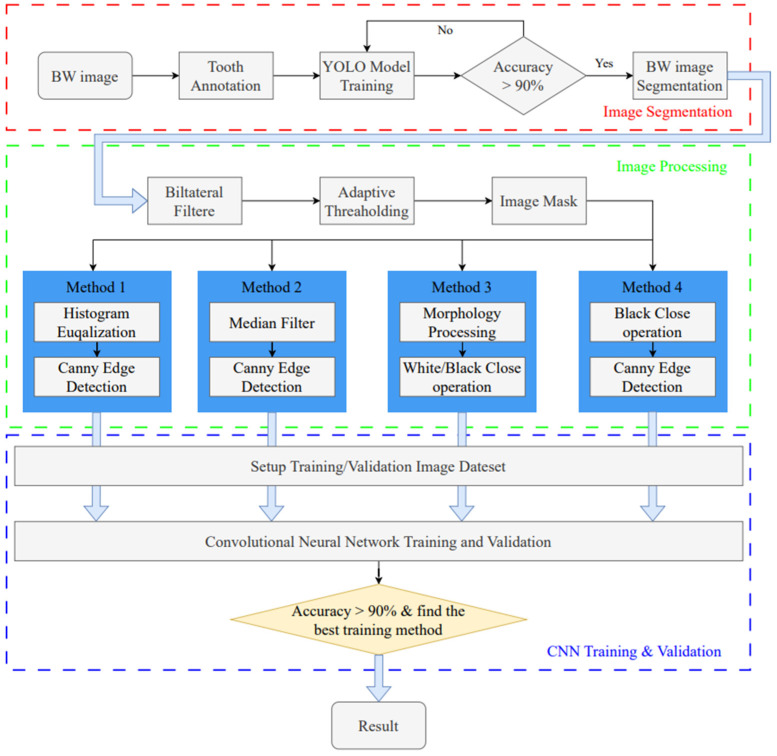
Intrabony defect assessment assisted-judgment flow diagram.

This study utilizes image enhancement and deep learning techniques to assess the severity of periodontal disease in BW images. The goal is to identify the intrabony defect of periodontal lesions and classify them into two categories: greater than 37 degrees and less than 37 degrees. The research process is divided into three stages, as shown in Figure 1. The first stage uses the YOLO object detection model to detect single tooth position and segments the BW image into individual tooth images. In the subsequent image processing stage, various image processing methods are applied to enhance the individual tooth image, aiming to find the most suitable image enhancement method for use in the CNN training and validation stage.

### 2.1. Data Collection

The BW image used in this study’s database was provided by Chang Gung Memorial Hospital in Taoyuan, Taiwan, with IRB approval: 202301730B0. A total number of 148 BW images were obtained from children aged 20–65 years in Taiwan. Among them, 82 are boys, and 66 are girls, and all BW images contain complete teeth. This study separates the dataset into an 80% training set and a 20% validation set for YOLO and the CNN training step, as shown in Table 1. Moreover, too few BW images in the dataset may lead to problems such as model overfitting and poor generalization ability. Many studies have employed data augmentation techniques to enhance the diversity of input features in X-ray images [9] and utilized cross-validation methods to mitigate overfitting and improve generalizability in small datasets [13]. This research achieved data augmentation by applying pepper noise, Gaussian filtering, ±5° rotation, and horizontal mirroring techniques, effectively increasing the dataset’s size and diversity. These methods resulted in an approximately threefold expansion of the dataset, ensuring the reliability of the model training process.

### 2.2. Image Segmentaion

BW images are particularly effective in capturing the alveolar crest. According to the literature [26], BW images maintain an orthogonal projection between the bone and teeth, reducing image distortion caused by improper shooting angles compared to periapical radiographs. In contrast, due to their curved projection, dental panoramic radiographs suffer from distortion and limit the observation of subtle changes in bone structure [27]. Therefore, this study utilizes the detailed view of gingival and dental structures provided by vertical BW images to assess the severity of intrabony defects. Since it is necessary to image the teeth and gingival symptoms to classify the degree of periodontal erosion using CNN, this study requires processing the image of a single gingival symptoms for CNN analysis. In this study, the YOLOv8 model will be trained, and the built-in segmentation function will be used to achieve a single gingival image.

#### 2.2.1. Annotation of Tooth

**Figure 2:**
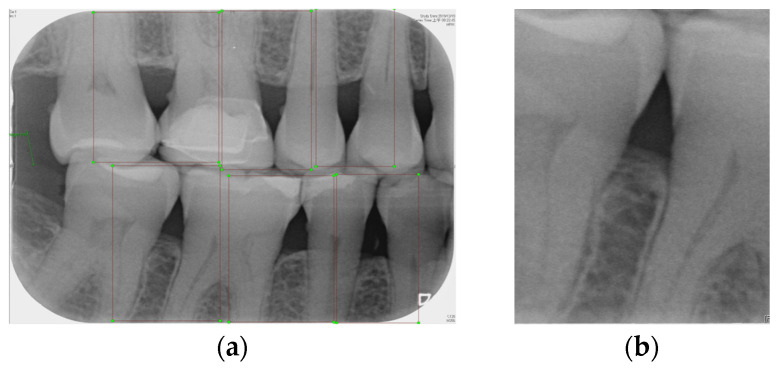
Image annotation and segmentation. (**a**) Image Annotation Diagram. (**b**) The tooth segmentation result.

Labeling was used as a tool, and by utilizing the rectangular tool, the marking range was from a single tooth section to another tooth section, which helps to accurately mark the actual condition of the gingiva around the middle of the tooth. Finally, the image was saved as a YOLO file and labeled as shown in Figure 2a. This process highlights the target area in the image and provides clearer information for CNN training.

#### 2.2.2. YOLOv8 Model Training

Numerous object detection models have been developed, such as the R-CNN series, Single Shot MultiBox (SSD), RetinaNet, and YOLO. Due to the complexity and variability in tooth alignment in each patient, the gums need to be separated at the BW film; YOLOv8 is the best model utilized in BW image segmentation for Angular Erosion. The YOLO model has an advantage in detecting the foreground and is highly efficient in considering the global information of the image and making judgments, and it also utilizes an anchor-free design. In the past, anchor frame detection was utilized to envelop various objects with predefined anchors. Regression adjustment was carried out through a series of operations, and the most accurate frames were retained using non-maximum suppression. Anchor-free detection predicts the center point of an object directly from the image to learn its location and size. This method helps detect objects with greater accuracy in various complex scenes, such as accurately locating teeth. Target detection of the vertical biting airfoil image is then performed by the trained YOLO model, as shown in Figure 2b.

### 2.3. Image Processing

Various steps will be taken for image symptom enhancement, including image processing before masking, specifically bilateral filtering and adaptive thresholding. After masking, image enhancement includes grayscale conversion, Gaussian filtering, histogram equalization, edge detection, closed operation, extracting the closed area, and filling. The multi-level enhancement highlights the symptomatic areas of the image, provides clear images and obvious contours to train the model, and improves the accuracy of the model.

#### 2.3.1. Bilateral Filter

**Figure 3:**
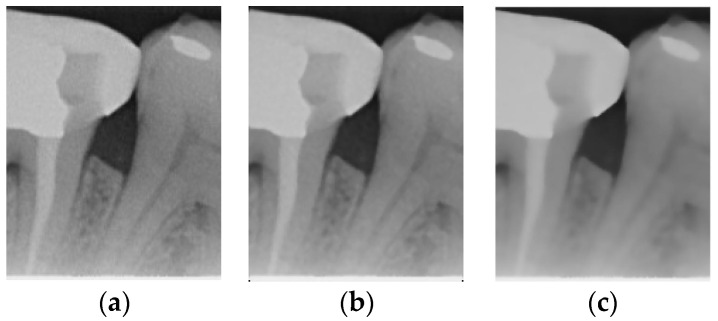
Comparison of filtering effects. (**a**) Original image. (**b**) Median filter. (**c**) Bilateral filter.

The original image has a lot of noise, which needs to be eliminated to smoothen the image. This study initially adopts median filtering, which is a non-linear digital filtering technique that is commonly used to remove noise. However, this method is not effective. In order to filter out the noise and preserve the edge details of the image at the same time, this study uses bilateral filtering (1) to solve this problem, which is shown in Figure 3.

(1) $$I_{bf}(p)=\frac{1}{W_{p}}\sum_{q\in S}I(q)q\cdot f_{s}(∥p-q∥)\cdot f_{r}(∥I(p)-I(q)∥)$$

where p and q denote the pixel positions in the image, and I(p) and I(q) denote the pixel values at positions p and q. S is a window centered at p, and the size of the window is controlled by a parameter. f_s_ is a weight function in the spatial domain, which is usually a Gaussian function, and denotes the effect of spatial distance. f_r_ is a weight function in the value domain, which is usually a Gaussian function, and denotes the effect of the pixel value difference. Wp is a normalization factor. It should be ensured that the weights sum to 1.

#### 2.3.2. Adaptive Thresholding

**Figure 4:**
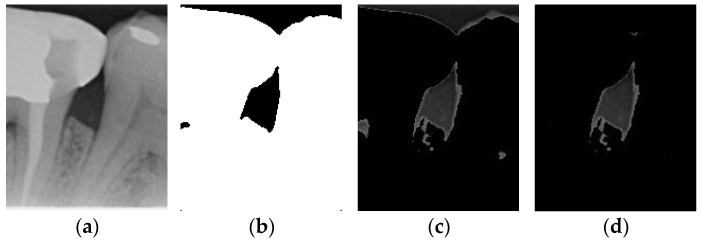
Comparison of thresholding images. (**a**) Original image. (**b**) Thresholding image. (**c**) The image applied to the original image by the negative effect. (**d**) Enhancement of Masked Image.

Image thresholding is carried out to extract the object contours or regions in the image for subsequent image analysis and processing. For the filtered image, this involves making the image contours more obvious and masking the unwanted regions. The adaptive threshold binarization is shown in Figure 4b, and it determines the threshold of each pixel point based on the distribution of pixel values within its neighborhood. Usually, the size and shape of the neighborhood are adjustable to better accommodate different image characteristics. This study adopts adaptive thresholding to superimpose the white regions in binarization onto the original image by using the negative effect after the excess teeth are binarized using adaptive thresholding for subsequent image enhancement, as shown in Figure 4.

#### 2.3.3. Enhancement of Mask

According to the above results, it is found that some images still have a few lines or color blocks in the non-symptomatic areas, which will affect the subsequent training of the model judgment. To solve this problem, the upper and lower 1/7 and the left and right 1/8 of the image are masked, which is the area where the symptoms are located; this is carried out not only to highlight the symptoms, but also to filter out the residual lines and color blocks in the non-symptomatic areas, which will be helpful for the subsequent steps. The result is shown in Figure 4d.

#### 2.3.4. Histogram Equalization and Edge Detection

**Figure 5:**
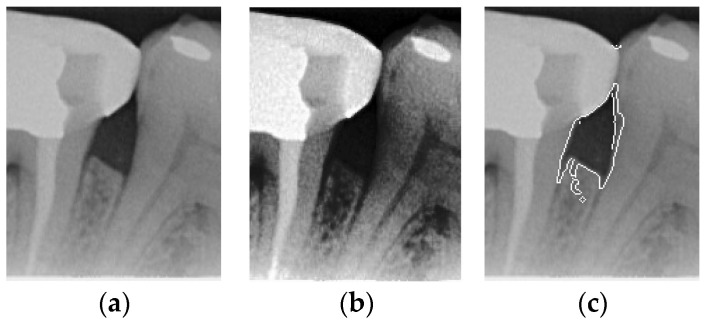
Comparison of Histogram equalization and Canny edge detection. (**a**) Original image. (**b**) Histogram equalization. (**c**) Canny edge detection.

Histogram equalization is often used to enhance the contrast and visual effect of an image by redistributing the pixel intensities of the image to make the gray level of the image more evenly distributed. Its advantages include simplicity and ease of implementation, and it can be used to enhance the overall contrast and brightness of the image; moreover, it does not require additional parameters, as shown in Figure 5b. Equation (2) is as follows, where L is the total number of gray levels in the image, p_r_(r) denotes the normalized histogram of gray levels in the input image, and T(r) denotes the transformation function of gray level r in the output image. In addition, Canny edge detection is also effective for symptom extraction. Canny is an algorithm based on gradient variation, in which non-extreme value suppression and double threshold design can obtain the accurate edge location, effectively depicting the highlighting of symptoms, as shown in Figure 5c.

(2) $$s=T\left(r\right)=\left(L-1\right)\sum_{j=0}^{r}p_{r}\left(j\right)$$

#### 2.3.5. Close Operation

**Figure 6:**
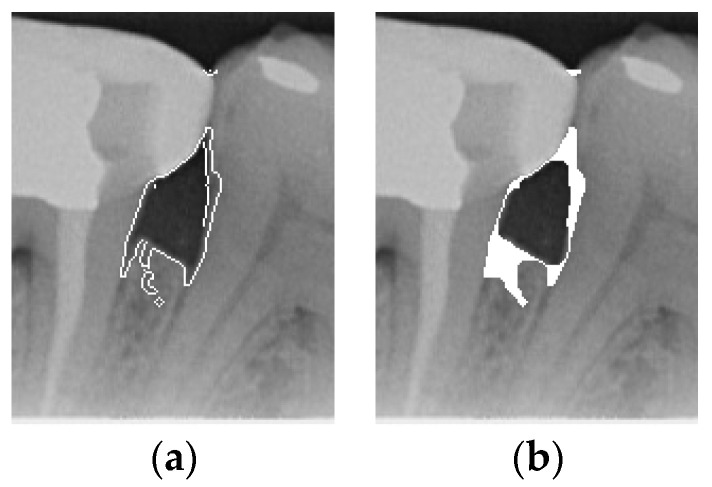
Comparison of Close operation. (**a**) Canny masked image. (**b**) Close operation.

According to the Canny operation image after masking, it is found that not every image can be depicted completely, even if all the symptoms are detected. There may be broken lines in the depiction, which may be caused by the different contrast and brightness of each image, resulting in a different depiction of each image. To solve this problem, the broken lines are compensated by the close operation to achieve the symptoms framed by the closed graphic for subsequent image processing as shown in Figure 6. Equation (3) is as follows:

(3) $$A\cdot B=\left(A⊕B\right)-B$$

#### 2.3.6. Extract Closed Area and Filling

**Figure 7:**
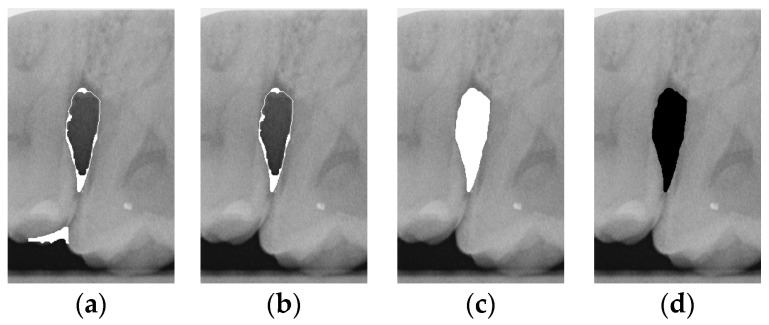
Comparison of extracting the closed area and filling. (**a**) Close operation. (**b**) Extracting the closed area. (**c**) Filling with white. (**d**) Filling with black.

To ensure that only the area framing the symptom is preserved, and to remove the rest of the interfering areas or lines, the symptom frames are extracted according to the closure algorithm, i.e., the maximum connectivity area can be preserved to remove the frames of the non-symptomatic areas, as shown in Figure 7b. After the closure process, some of the frame lines are less smooth than others, which may affect the discrimination accuracy. To deal with this situation, this study performs the filling of the frame lines at the symptom area to facilitate the training of the model. Black and white are chosen to highlight the contrast and to compare the difference in discrimination between the two fill colors, as shown in Figure 7c,d.

### 2.4. CNN Training and Validation

In automatic medical image detection systems, deep learning technology has become an indispensable tool, with convolutional neural networks (CNN) being particularly prominent due to their excellent capabilities in processing image data. Through a series of convolutional layers, these networks can capture and extract various features in an image, from low-level edges to high-level complex structures, achieving a deep understanding of the image. The architectural design of CNNs makes them extremely efficient when processing images. The convolution layer uses convolution kernels to scan the image and extract local features, reducing the number of parameters. The pooling layer is then used to further reduce data dimensions and computational complexity while retaining key features. This multi-level feature extraction process enables CNNs to effectively learn and identify important information in images. Transfer learning further improves the application efficiency of deep learning in medical image analysis. Transfer learning leverages models pre-trained on large datasets, applying the features and knowledge learned in these models to new tasks. It not only saves a significant amount of training time and computing resources but also significantly improves the model’s generalization ability. Additionally, the advantages of transfer learning include rapid and strong adaptability to new tasks, versatility, and high performance. By using pre-trained models, this study can quickly apply the rich features learned by these models to large-scale image data, better addressing the challenges in medical image analysis and achieving excellent results. This study selected five CNN models for transfer learning, which are widely used in the field of image recognition and classification. By comparing and evaluating these models, we hope to identify the one that performs best in terms of accuracy, thereby improving the reliability and accuracy of clinical symptom identification to help medical professionals make more accurate decisions in diagnosis and treatment. To train the CNN model and object detection model, this study utilized an Nvidia GeForce RTX 3060 GPU to accelerate the training of the deep learning model, as shown in Table 2.

To optimize the performance of the model to the greatest extent, this study implemented the following strategies. The first is hyperparameter adjustment, which optimizes the model training process by adjusting different combinations of hyperparameters, such as learning rate, batch size, number of iterations, etc. These settings help improve the convergence speed and performance of the model, making it more suitable for specific disease symptom identification tasks. L2 regularization was applied to prevent overfitting by adding a penalty term to the loss function, which limits the size of the model parameters. This technique prevents the model from relying too heavily on noise in the training data by ensuring that parameter values remain small, thereby maintaining model stability and smoothness. The last is indicator evaluation. During the model training process, the trained model’s accuracy, loss function, and recall rate are used to evaluate the model’s performance in different aspects. This assists in optimizing the model and adjusting its hyperparameters to more comprehensively improve the training process.

#### 2.4.1. CNN Structure

This study uses five different CNN models: MobileNet, EfficientNet, InceptionNet_v3, XceptionNet, and ResNet50. Taking MobileNet as an example, the structure is shown in Table 3. The MobileNet model combines a multi-layer network, including multiple convolutional layers, deep convolutional layers, max-pooling layers, and fully connected layers. The convolutional layer and the deep convolutional layer are used to extract image features, while the max pooling layer is used for downsampling to reduce the size of the feature map, thereby decreasing computational load and controlling overfitting. The fully connected layer is responsible for the final classification decision. ReLU is extensively used in the network layers as the activation function. ReLU introduces nonlinearity, allowing the neural network to better approximate complex functions and patterns, improving the model’s performance. ReLU effectively addresses the vanishing gradient problem during backpropagation, which is often caused by traditional activation functions such as Sigmoid and Tanh. By setting some of the output neurons to zero, ReLU increases the network’s sparsity, alleviates overfitting, and enhances the model’s generalization ability. To match the input image size of the CNN, the input size is adjusted to 227 × 227 × 3 pixels. In the fully connected layer, the classification task only requires distinguishing the target into two categories, and the original output size of 1000 is adjusted to 2. This modification meets the needs of the classification tasks.

#### 2.4.2. Hyperparameters

MobileNet’s depth-separable convolution technology has clear advantages in terms of computational efficiency and resource requirements. Its lightweight design also makes it easier to deploy on various hardware platforms and is suitable for clinical application scenarios that require real-time processing. Therefore, MobileNet is chosen as the first CNN model studied. This study first obtained the optimal hyperparameter settings through MobileNet training, as shown in Table 4. These settings are uniformly applied to other CNN models. This approach prevents subsequent model comparisons from being unreliable because of differing conditions and allows each model to be fairly compared under the same hyperparameter settings, ensuring the determination of the best-suited model.

In machine learning and deep learning, the initial learning rate, the maximum number of iterations (Max Epoch), and the mini-batch size are key hyperparameters that directly affect the training process and final performance of the model. This study conducted multiple experiments on the initial learning rate, ranging from 0.01 to 0.00001, and finally selected 0.0001 as the most stable and least variable initial learning rate. The maximum number of iterations (Max Epoch) indicates the number of times the model is fully trained on the entire training dataset. A sufficient number of iterations helps the model fully learn the features in the training data and improves model performance.

Mini-batch size refers to the size of the training data subset used each time the model parameters are updated. Choosing an appropriate mini-batch size can strike a balance between computational efficiency and the smoothness of gradient estimates. This study used a range between 2 and 32 for training when setting the mini-batch size and found that the optimal mini-batch size was 16. Therefore, choosing the appropriate initial learning rate, maximum number of iterations, and mini-batch size improves the training stability and enhances the generalization ability of the model.

#### 2.4.3. CNN Training

After training the CNN model, five key indicators—accuracy, precision, recall, F1 score, and confusion matrix—are used to evaluate its performance, as outlined in Equations (4)–(7) and Table 5. The confusion matrix compares predicted values with actual values, presenting results in terms of True Positive (*TP*), True Negative (*TN*), False Positive (*FP*), and False Negative (*FN*). *TP* indicates correct predictions for positive cases, *TN* for correct predictions of negative cases, *FP* for incorrect positive predictions, and *FN* for incorrect negative predictions. These indicators collectively assess the model’s quality to ensure its effectiveness in practical applications.

(4) $$Accuracy\;=\;\frac{TP+TN}{TP+TN+FP+FN}$$

(5) $$Precision=\frac{TP}{TP+FP}$$

(6) $$Recall=\frac{TP}{TP+FN}$$

(7) $$F1\;Score=2\times \frac{precision\times recall}{precision+recall}$$

## 3. Results

In this section, this study will analyze the experimental results and compare them with advanced research. This section can be divided into tooth detection, various image enhancements, and post-detection results.

### 3.1. Image Segmentation

**Figure 8:**
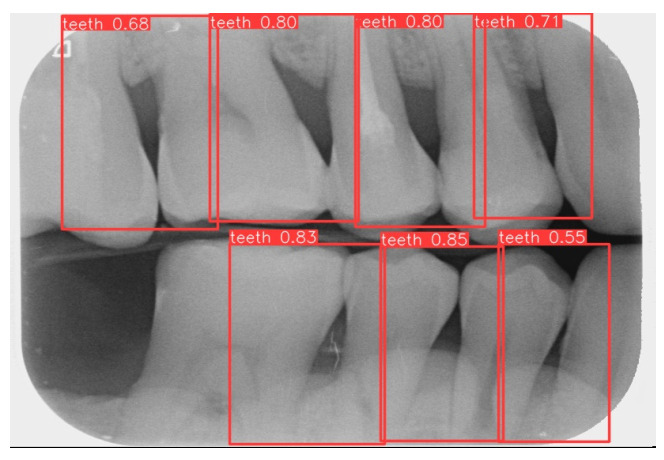
YOLOv8 detection result.

This study uses YOLOv8 to detect the gingiva between each tooth and record its coordinates. The parameters are listed in Table 6. The final target detection results of this study are shown in Figure 8. The comparison between the YOLO detection model and other studies is presented in Table 7. The image shows that the gum features are completely preserved, providing a reliable basis for CNN.

### 3.2. CNN Training Result

**Figure 9:**
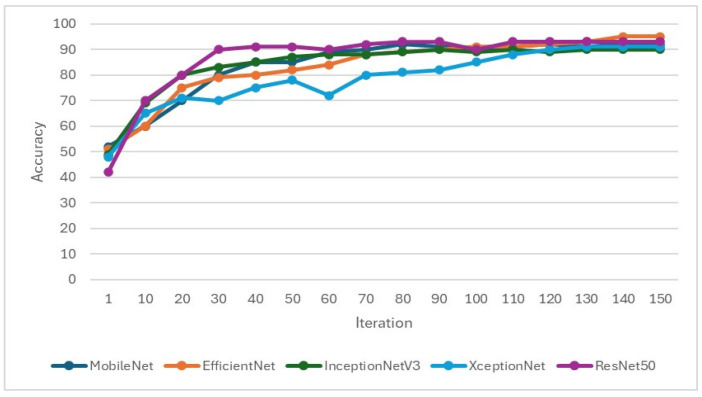
Validation accuracy during the training process of the CNN model.

**Figure 10:**
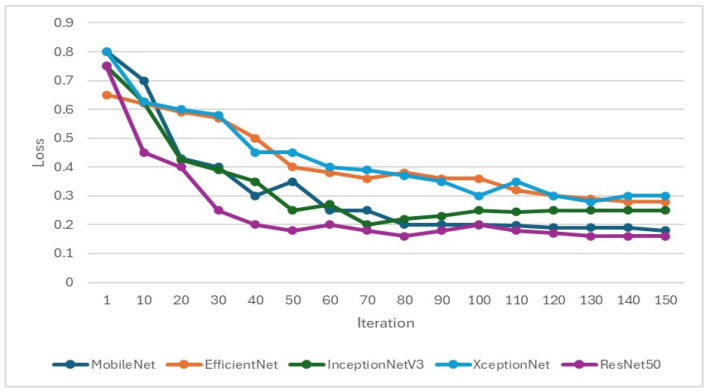
Validation loss function during the training process of the CNN model.

In the training of the CNN model, it is important to focus on whether the accuracy is effectively improving and monitor whether the loss function decreases when training. If the loss function does not decrease or even increase while the accuracy increases, it may indicate overfitting. Therefore, when setting hyperparameters for CNN training, L2 regularization will be added to overcome model overfitting, as shown in Figure 9 and Figure 10. Hyperparameters and feature enhancement content should be adjusted by observing changes in model accuracy and the loss function.

This study selected EfficientNet and MobileNet as primary CNN architectures due to their superior performance in preliminary experiments. Specifically, Efficient-Net achieved an accuracy of 78.85% with an 8:2 training–validation split, significantly outperforming the 55.06% accuracy obtained with a 7:3 split. Similarly, MobileNet reached an accuracy of 84.62% using the 8:2 split, compared to 80.38% with the 7:3 split. These results indicate that an 8:2 split, which provides more data for training, enhances the models’ learning capacity and generalization ability. Thus, this study adopted the 8:2 training–validation split across all models to optimize performance.

For the symptoms of periodontal lesions, this study uses feasible image enhancement techniques to enhance the features of the symptoms and combines multiple enhancements to find the model with the best training effect. According to the enhancement part of image processing, this study employs four methods, namely edge processing, median filtering, closed operation filling, and line detection to perform impact processing, and explores the most suitable method through CNN training.

In the first method, since the goal of this study is to use CNN to determine whether the intrabony defect of periodontal disease intrabony defects is greater than or less than 37 degrees, two different enhancement techniques of the Canny algorithm are used: histogram equalization and the Canny algorithm method for feature enhancement. The results are shown in Table 8. As the data indicates, the model using the Canny algorithm performs better. Compared with histogram equalization, the accuracy of the validation set can be increased by 2.88% to 15.54%. Therefore, the Canny algorithm is used to proceed to the next step of image processing.

In the second method, this study attempts to use a combination of median filtering and the Canny algorithm for training. It compares the training results of median filtering alone with those of median filtering combined with the Canny algorithm. The results are shown in Table 9. The study finds that coupling the Canny algorithm with different models does not effectively improve the validation accuracy of all models. Therefore, further discussion is needed to determine the most suitable image processing method.

The third method employs morphological closing operations for image processing. Although the Canny algorithm can outline the edges of the teeth and eliminate noise, some noise remains unremoved, and the depicted edges are not continuous and complete, leading to inaccuracies in lesion depiction. Therefore, this study tests three different approaches to morphological closing: standard closing, closing with black fill, and closing with white fill. The results are shown in Table 10. However, after training, it is found that the effects of filling with black and white are not better than the original closing operation, and there is no significant improvement in validation accuracy. Thus, further exploration of different methods is needed to find a more suitable image processing approach.

The fourth method builds on previous experience with closed operation filling and the Canny algorithm. The Canny algorithm is applied after the closed operation filling, allowing the edges drawn by the closed operation filling to more clearly fit the lesion. This new detection line algorithm is then compared with the closed operation filling method, as shown in Table 11. The results indicate that the enhanced detection line algorithm can significantly improve the verification accuracy, with improvements ranging from 1.22% to 7.31%. Notably, the verification accuracy of EfficientNet reaches 95.12%, making it the CNN with the best training effect in this study. The confusion matrix in Table 12 shows the model’s prediction accuracy for the two categories of “Angle ≤ 37°” and “Angle > 37°”, indicating relatively high accuracy with a few misclassifications. Following that, the model indicators in Table 13 reveal that the model performed excellently in Precision, Recall, and F1-Score across the two categories (“Angle ≤ 37°”, “Angle > 37°”), with F1-Scores all above 94%. Finally, the image validation results in Table 14 demonstrate the model’s accuracy across different angle ranges using actual images, highlighting the model’s high consistency and accuracy in practical applications for periodontal angle classification. Collectively, these results indicate that the EfficientNet model exhibits strong predictive performance and stability in periodontal image classification.

## 4. Discussion

This study focuses on the automated detection of the radiographic defect angle of the intrabony defect and its application as an auxiliary diagnostic technology for periodontal bone loss in various medical institutions. In traditional periodontal disease detection, research [30] utilized a digital image processing system to classify periodontal disease, achieving a maximum accuracy of approximately 90%. Furthermore, ref. [31] highlighted that vertical BW images are more effective than horizontal BW images for assessing periodontal bone loss. With advancements in artificial intelligence, there has been increasing research on AI-assisted periodontal disease diagnosis. For instance, ref. [28] employed the YOLOv8 object detection model for BW image segmentation, replacing traditional feature-based segmentation methods. Using YOLOv8, the model achieved a precision of 76% in identifying teeth affected by periodontal disease, with an F1 score improvement of 15.2% compared to YOLOv4 in detecting alveolar bone loss. Additionally, AlexNet [29] achieved an accuracy of 88.8% in detecting tooth positions from X-ray images. One study [32] demonstrated that the precision of AI models for periodontal disease detection could reach up to 81%. In summary, most current studies use AI models to detect tooth positions in periodontal disease, with a maximum accuracy of approximately 90%. This study used object detection technology for tooth detection, with YOLOv8 as the tooth detection model, achieving a maximum accuracy of 94.32%. Compared to existing studies, this represents an improvement of approximately 4.32% in detection accuracy.

Furthermore, while methods such as YOLOv8, EfficientNet, and image enhancement techniques are well-established, the novelty of this study lies in its focus on using intrabony defect angles for periodontal diagnosis and treatment planning—a critical yet underexplored application. This study bridges a significant gap in clinical practice by providing an automated, quantitative tool to assist dentists in evaluating intrabony defects, reducing reliance on subjective assessments, and improving the accuracy and efficiency of periodontal diagnosis. Current research on grading the radiographic defect angle of the intrabony defect is limited, and most are limited to algorithm design and evaluation [30]. No system uses artificial intelligence to assist dentists in diagnosing and treating intrabony defects. Thus, this study developed a novel AI-assist method for identifying the severity of periodontal disease by categorizing it into average, mild loss, and severe loss based on a 37-degree angle. Unlike studies that identify periodontal disease, this approach introduces a different methodology for medical image detection.

This study analyzes various image processing methods to improve the accuracy of CNN verification. Various image enhancement techniques were applied and evaluated using five well-established CNN models for classification. The experimental results demonstrated that EfficientNet achieved the best validation accuracy of 95.12% when combined with the Canny algorithm. This study also explored the impact of different image enhancement techniques on CNN-based image classification. Among the methods compared, combining morphological closing operations with Canny edge detection resulted in the most significant improvement in accuracy, achieving an enhancement of 16.27%. Enhancing the edge features of periodontal lesions played a crucial role in improving the model’s detection accuracy. In contrast, median filtering and Canny edge detection did not significantly improve validation rates, nor did morphological closing operations, including black-and-white filling, lead to notable accuracy gains. Ultimately, this study found that the fourth image enhancement method (Close algorithm combined with Canny Edge detection) can maximize the accuracy of CNN in classifying intrabony defects and improving validation accuracy by 7.31%, demonstrating the effectiveness of this approach. Furthermore, compared to the state-of-the-art AI technique used for determining whether a tooth is affected by periodontal disease [33], which achieved a precision of 82.43%, the method proposed in this study improved precision by approximately 13.4%, as shown in Table 15. Additionally, this approach offers a grading system for periodontal disease severity, providing dentists with an alternative tool for auxiliary diagnosis and treatment planning. The primary contributions of this study are as follows:

1. This study first introduces a 37-degree angle-based method for classifying the severity of periodontal disease intrabony defect angle symptoms, offering an alternative approach to medical image detection.
2. The close algorithm combined with Canny Edge detection improved accuracy by up to 7.31% with EfficientNet reaching 95.12% accuracy.
3. Using a multi-step feature enhancement process instead of single filtering, combining methods to achieve optimal results, can improve the 13.4% mean accuracy.

However, this study faces several challenges and limitations, including data privacy concerns and potential biases in the model. Due to data privacy considerations, the dataset used in this study was relatively small, which limited the model’s generalization capability and robustness. This may lead to instability in the model’s performance when applied in real-world settings. Additionally, potential sample distribution biases in the dataset (e.g., uneven patient age distribution, gender, or lesion characteristics) may affect the model’s fairness and applicability. To address the challenges associated with a small dataset, various studies have utilized data augmentation techniques to increase the diversity of input features from X-ray images and cross-validation methods to reduce overfitting and enhance generalizability. This research applied pepper noise and Gaussian filtering, ±5° rotation, and horizontal mirroring techniques to augment the dataset and improve feature diversity. Additionally, the results were validated using five distinct CNN models with cross-validation to ensure the precision of image classification, even with a limited dataset size. Future studies are encouraged to employ more extensive and diverse datasets, combined with multi-center data, to further enhance the model’s generalizability and robustness, thereby supporting broader clinical applications.

In clinical practice, implementing this technology also faces several specific challenges. Firstly, most dental clinics have already established fixed diagnostic and treatment workflows. Integrating AI technology into these workflows may require adjustments to existing hardware and software systems and training personnel, potentially impacting initial adoption and application efficiency. Moreover, deploying AI technology may involve additional hardware requirements (such as high-performance computing devices) and software development costs, which could pose financial burdens for smaller dental clinics. Additionally, the costs of maintenance and updates must be considered to ensure the long-term stability and accuracy of the system. In future work, to address these issues, this study envisions integrating the intrabony defect classification model into digital imaging workflows (such as bitewing or CBCT scans), enabling direct data acquisition from radiographic systems. The model’s classification results can support treatment planning through image enhancement and lesion detection techniques, such as guided tissue regeneration (GTR) or other regenerative techniques. Furthermore, by integrating the model’s outputs with existing dental management systems (DMS), clinical workflows can be optimized to improve diagnostic efficiency and reduce the workload of dentists.

## 5. Conclusions

This research developed a detection line algorithm combined with CNN technology and applied it to medical image detection systems. The proposed system improves the accuracy of detecting the radiographic defect angle of the intrabony defect of periodontal lesions in BW images. By automating the classification of the radiographic defect angle of the intrabony defect, this intrabony defect detection system aims to assist dentists in diagnosing and assessing the severity of periodontal disease more accurately. It also reduces the risk of human error and helps clinicians address patients’ oral health issues quickly and effectively, ultimately contributing to improved treatment outcomes. Building on the current research findings, future studies could expand the system’s functionality and application in several directions. For instance, incorporating a model to determine the presence or absence of periodontal disease could provide a more comprehensive diagnostic tool. Integrating multi-modal image data, such as CBCT or panoramic radiographs, may enhance diagnostic accuracy. Developing specialized models tailored for different age groups could also improve the system’s applicability to diverse patient populations. Moreover, as part of future work, this study plans to expand the dataset by collecting more PA images, which are provided to improve the model’s robustness and reliability. However, it is important to note that open-source datasets specifically targeting intrabony defects of periodontal lesions in PA images are currently not publicly available, which presents a challenge for immediate external validation. While this system demonstrates promising results in periodontal disease detection, further refinement and validation are necessary to realize its full potential. By continuing to innovate and collaborate, this research contributes to advancements in dental medicine and medical imaging technologies, ultimately aiming to improve the quality of oral health care and patient outcomes on a broader scale.

## Figures and Tables

**Table 1:** The image dataset used in YOLO object detection and CNN classification.

**BW image dataset after data augmentation used for YOLO object detection**						
Training Set		Validation Set			Total	
75		375			450	
**Single tooth image dataset used for CNN classification**						
	Original		Augmentation	Training Set		Validation Set
(intrabony angle < 37)	58		174	46		12
(intrabony angle > 37)	74		222	59		15
Total	132		396	105		27

**Table 2:** Hardware and software platform.

**Hardware Platform**	**Version**
CPU	12 Gen Intel(R) Core(TM) i7-12650@2.30GHz
GPU	NVIDIA GeForce RTX 3050 8G
**Software platform**	**version**
MATLAB	R2021b
Python	3.10.8

**Table 3:** MobileNet structure.

Layer	Filters	Filter Size	Stride	Padding	Channel Size	Activation Functions
Input					227 × 227 × 3	
Conv1	32	3 × 3	2	1	114 × 114 × 32	ReLU
Depthwise Conv1	32	3 × 3	1	1	114 × 114 × 32	ReLU
Pointwise Conv1	64	1 × 1	1	0	114 × 114 × 64	ReLU
MaxPool1	-	2 × 2	2		57 × 57 × 64	
Depthwise Conv2	64	3 × 3	2	1	28 × 28 × 64	ReLU
Pointwise Conv2	128	1 × 1	1	0	28 × 28 × 128	ReLU
Depthwise Conv3	128	3 × 3	1	1	28 × 28 × 128	ReLU
Pointwise Conv3	128	1 × 1	1	0	28 × 28 × 128	ReLU
MaxPool2	-	2 × 2	2		14 × 14 × 128	
Depthwise Conv4	128	3 × 3	2	1	14 × 14 × 128	ReLU
Pointwise Conv4	256	1 × 1	1	0	14 × 14 × 256	ReLU
Depthwise Conv5	256	3 × 3	1	1	14 × 14 × 256	ReLU
Pointwise Conv5	256	1 × 1	1	0	14 × 14 × 256	ReLU
MaxPool3	-	2 × 2	2		7 × 7 × 256	
Depthwise Conv6	256	3 × 3	2	1	7 × 7 × 256	ReLU
Pointwise Conv6	512	1 × 1	1	0	7 × 7 × 512	ReLU
Depthwise Conv7	512	3 × 3	1	1	7 × 7 × 512	ReLU
Pointwise Conv7	512	1 × 1	1	0	7 × 7 × 512	ReLU
Global Avg Pooling	-	7 × 7			1 × 1 × 512	
FC	2				1 × 1 × 2	
Softmax	-				1 × 1 × 2	Softmax
Output	-				1 × 1 × 2	

**Table 4:** CNN hyperparameters setting.

Hyperparameter	Value
Mini-Batch Size	16
Initial Learning Rate	0.0001
Max Epochs	50
Shuffle	Every-epoch
Validation Data	imgTest
Validation Frequency	5
Execution Environment	GPU
Verbose	FALSE
Plots	Training-progress
Learning Rate Schedule	Piecewise
Learning Rate Drop Factor	0.01
Learning Rate Drop Period	10
Validation Patience	5

**Table 5:** Confusion matrix.

	Positive	Negative
Positive	True Positive	False Negative
Negative	False Negative	True Negative

**Table 6:** YOLOv8 training parameters.

Hyperparameter	Value	Hyperparameter	Value
Epochs	200	Lr0	0.01
Imgsz	640	Lrf	0.01
Batch	8	Patience	10

**Table 7:** Comparison of YOLOv8 in single tooth detection with other dissertation models.

Metrics	This Study Method	Method in [28]	Method in [29]
	YOLOv8	YOLOv5	AlexNet
Precision	94.32%	76%	88.8%
Recall	94.4%	83.42%	92.84%
mAP50	95.5%	No data	No data
F1 Score	91.2%	76%	No data

**Table 8:** CNN training results after Canny algorithm compared to the original image.

		MobileNet	EfficientNet	InceptionV3	XceptionNet	RestNet
Original	Accuracy	84.62%	78.85%	85.58%	74.04%	84.62%
	Training time	2 min 3 s	7 min 12 s	1 min 53 s	16 min 25 s	1 min
Histogram Equalization	Accuracy	90.62%	72.92%	91.67%	75%	86.54%
	Training time	3 min 49 s	5 min 31 s	2 min 52 s	16 min 30 s	1 min 36 s
canny	Accuracy	89.58%	91.67%	92.71%	89.58%	87.50%
	Training time	2 min 21 s	5 min 5 s	2 min 52 s	12 min 9 s	1 min 21 s

**Table 9:** Results of CNN training with median filtering and overlay Canny algorithm.

		MobileNet	EfficientNet	InceptionV3	XceptionNet	RestNet
Median	Accuracy	93.75%	93.75%	87.50%	84.38%	89.58%
	Training time	3 min 12 s	9 min 31 s	1 min 31 s	9 min 33 s	1 min 22 s
Canny + Median	Accuracy	91.67%	92.71%	84.38%	88.54%	93.75%
	Training time	5 min 31 s	5 min 56 s	1 min 44 s	10 min	1 min 27 s

**Table 10:** CNN training results in close operation filling in black and filling in white.

		MobileNet	EfficientNet	InceptionV3	XceptionNet	RestNet
Closing	Accuracy	91.46%	89.02%	82.93%	84.15%	89.02%
	Training time	1 min 59 s	7 min 10 s	1 min 47 s	10 min 45 s	1 min 13 s
Closing black	Accuracy	92.86%	85.71%	89.29%	78.57%	90.48%
	Training time	2 min 53 s	4 min 8 s	1 min 38 s	12 min 27 s	1 min 20 s
Closing white	Accuracy	90.48%	88.10%	86.90%	83.33%	89.29%
	Training time	2 min 51 s	7 min 38 s	1 min 23 s	17 min 41 s	1 min 17 s

**Table 11:** CNN training results after close operation, filling, and applying the Canny algorithm.

		MobileNet	EfficientNet	Inception_v3	XceptionNet	RestNet
Closing	Accuracy	91.46%	89.02%	82.93%	84.15%	89.02%
	Training time	1 min 59 s	7 min 10 s	1 min 47 s	10 min 45 s	1 min 13 s
Close Edge detection	Accuracy	92.68%	95.12%	90.24%	91.46%	93.90%
	Training time	2 min 43 s	6 min 49 s	2 min 39 s	9 min 57 s	45 s

**Table 12:** Confusion matrix for EfficientNet training models.

		Actual	
		Angle < 37	Angle > 37
Predicted	Angle < 37	42	3
	Angle > 37	1	36

**Table 13:** EfficientNet training model indicator.

	Angle < 37	Angle > 37	Average
Precision	97.67%	92.31%	95.25%
Recall	93.33%	97.30%	95.12%
F1-Score	95.45%	94.73%	95.18%

**Table 14:** Periodontal image validation with 5-fold CNN validation.

Ground Truth Angle < 37	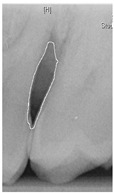	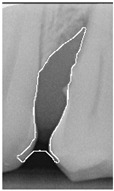	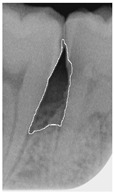	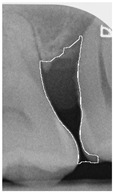
Accuracy	94.80% angle < 37	97.53% angle < 37	98.13% angle < 37	97.65% angle < 37
Ground Truth Angle > 37	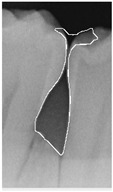	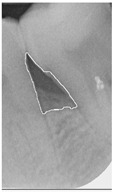	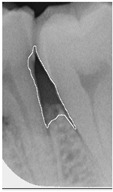	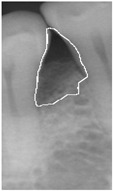
Accuracy	96.19% angle > 37	96.30% angle > 37	99.36% angle > 37	98.37% angle > 37

**Table 15:** Comparison with other disease classification methods.

Method	This Study Used EfficientNet			YOLOv8-cls [33]
	Before Enhancement	Histogram Equalization	Close Canny Edge Detection	
Accuracy	78.85%	72.92%	95.12%	77.03%
Precision	83.45%	84.66%	95.25%	82.43%
Recall	79.66%	82.17%	95.12%	71.62%

## Data Availability

We have provided the algorithm and image classification model developed in this study, as well as the research results on GitHub, to facilitate further research by other researchers. The GitHub repository can be accessed at: https://github.com/jojowang234/bioengineering3342859 (accessed on 31 December 2024). The dataset used in this study was approved by the Institutional Review Board (IRB) for ethical compliance. However, the dataset cannot be publicly available due to confidentiality agreements. Despite this limitation, the shared GitHub resources allow for replicating and extending the study’s methodology. The GitHub repository includes the following components: YOLO model folder: This folder predicts and segments teeth using a pre-trained model (yolov8n). The model loads the pre-trained weights in the training code, processes the specified source image for segmentation, and saves the results in a designated project folder. The source parameter specifies the image to be segmented, the project parameter defines the folder name for saving the results, and the name parameter determines the names of cropped images after segmentation. Image Processing folder: The image processing workflow is divided into two stages: pre-processing and processing. Pre-Processing: Images are enhanced using bilateral filtering, binarization, masking, grayscale conversion, histogram equalization, Gaussian filtering, and Canny edge detection. Processing: Enhanced images undergo further operations, including masking, pixel value adjustment, morphological closing, color filling, preservation of the maximum connectivity region, and superimposing the processed image onto the original image. CNN folder: This section contains five CNN models used for classification. Testing: After training, each class’s precision, recall, and F1 scores are calculated to evaluate performance. Params: The repository includes pre-trained parameters for the five CNN models and the test data generated after training. Testing Program: This program classifies user-provided images by returning the most likely class and its probability. By sharing these resources, we aim to enable researchers to replicate, build upon, and extend our work, fostering advancements in this area while maintaining ethical compliance and protecting the confidentiality of the dataset.
